# Supplementary material for: Closing the Gap in Proteomic Identification of Histoplasma capsulatum: A Case Report and Review of Literature
Source: J Fungi (Basel). 2023 Oct 15;9(10):1019. doi: 10.3390/jof9101019 (PMC10607645; doi:10.3390/jof9101019)
Supplement: Supplementary file 1 [file jof-09-01019-s001.zip › jof-2595986-supplementary.pdf]

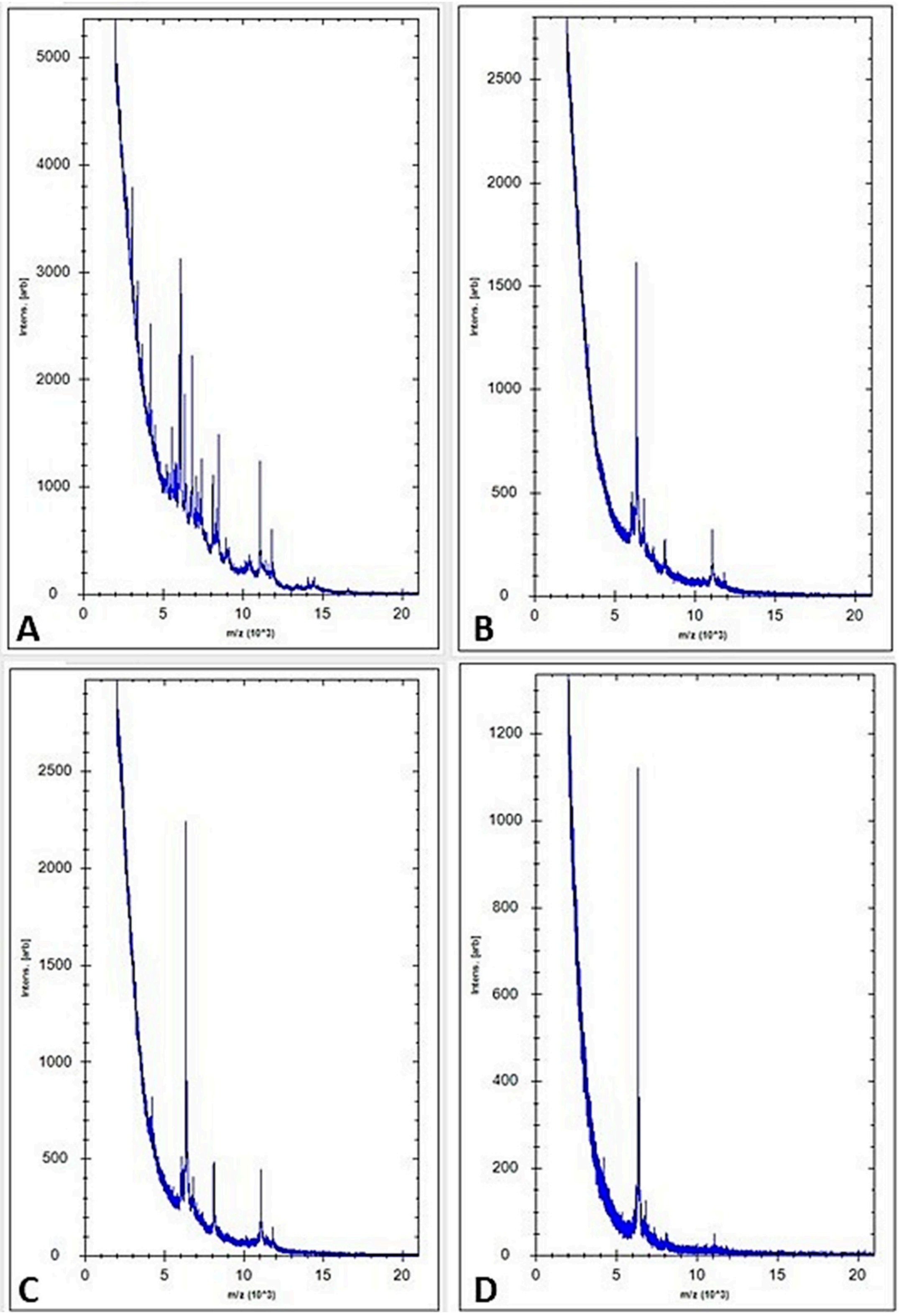

Figure S1. MALDI-TOF spectra analyses from fungal culture on SDA at 25°C. The obtained peaks, corresponding to a low log score, show a misidentification of *H. capsulatum* as other fungal pathogens and, specifically, with A) *Aspergillus niger* (1.15); B) *Talaromyces rugulosus* (1.05); C) *Microsporum audouinii* (1.07) and D) *Chrysosporium shanxiense* (1.14).

```
Query  1     TGTGAAACTGTTGAAAGGGAAGCGCTTGCGACCAGAGTCGGCCGCGGGGTTTCAGCGGGC  60
             ||||||| ||||||||||||||||||||||||||||||||||||||||| ||||||||||
Sbjct  929   TGTGAAATTGTTGAAAGGGAAGCGCTTGCGACCAGAGTCGGCCGCGGGGGTTCAGCGGGC  988

Query  61    ATTCGTTGCCCGTGCAATCCCCCGCGGCCGGGCCAGCGTCGGTTTCGACGGCCGGTCAAA  120
             ||||||||||||||||||||||||||||||||||||||||||||||||||||||||||||
Sbjct  989   ATTCGTTGCCCGTGCAATCCCCCGCGGCCGGGCCAGCGTCGGTTTCGACGGCCGGTCAAA  1048

Query  121   GGCCCCCGGAATGTGTCGCCTCTCGGGGCGTCTTATAGCCGGGGGTGCAATGCGGCCAGT  180
             ||||||||||||||||||||||||||||||||||||||||||||||||||||||||||||
Sbjct  1049  GGCCCCCGGAATGTGTCGCCTCTCGGGGCGTCTTATAGCCGGGGGTGCAATGCGGCCAGT  1108

Query  181   CGGGACCGAGGAACGCGCTCCGGCACGGACGCTGGCTTAATGGTCGTCAGCGACCCG  237
             |||||||||||||||||||||||||||||||||||||||||||||||||||||||||
Sbjct  1109  CGGGACCGAGGAACGCGCTCCGGCACGGACGCTGGCTTAATGGTCGTCAGCGACCCG  1165
```

Figure S2. Sequencing analysis results by PCR-amplified DNA. Identities 235/237 (99%); gaps 0/237 (0%).

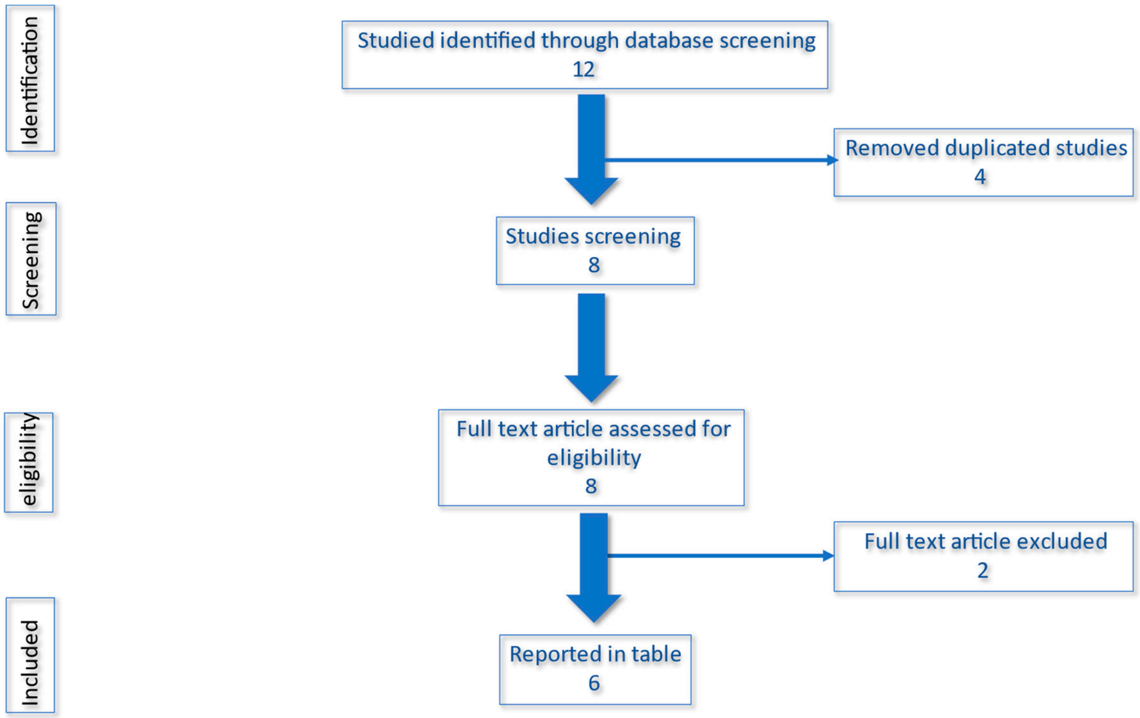

Figure S3. Flow chart reporting the data selection and analysis method used in the systematic review. Twelve articles including the application of MALDI-TOF technology for *H. capsulatum* identification were examined. Of these, four were excluded after the application of exclusion criteria (articles discussing proteomic methods other than MALDI-TOF and non-English language publications). Among the eight remaining publications eligible for analysis, two were excluded after reading the abstract or the full text (did not concern cases of identification of *H. capsulatum* by MALDI in human). Six articles were included in the study.
